# Influence of Roll Speed during Roll Compaction and Its Effect on the Prediction of Ribbon Solid Fraction

**DOI:** 10.3390/pharmaceutics14112399

**Published:** 2022-11-07

**Authors:** Martin Lück, Matthias De Saeger, Peter Kleinebudde

**Affiliations:** 1 Institute of Pharmaceutics and Biopharmaceutics, Heinrich Heine University Duesseldorf, Universitaetsstrasse 1, 40225 Duesseldorf, Germany; 2 Laboratory of Pharmaceutical Technology, Department of Pharmaceutics, Ghent University, Ottergemsesteenweg 460, 9000 Ghent, Belgium

**Keywords:** roll compaction, roll speed, critical quality attribute, solid fraction, granule size, tabletability, Midoux number, solid fraction prediction

## Abstract

Influence of the roll speed (*RS*) during roll compaction on ribbon, granule, tablet properties and its effect on the prediction of the ribbon solid fraction at-gap is often neglected or controversially discussed. The aim of this study was to investigate the effect of the *RS* systematically. Microcrystalline cellulose (*MCC*) and lactose were compressed at several maximum roll pressures ($P_{max}$) and *RS* combinations using a gap-controlled roll compactor. The ribbon solid fraction after elastic recovery ($SF_{ribbon}$), granule size distribution and tabletability of the granules as well as the ribbon solid fraction at-gap $\left(SF_{gap}\right)$ were measured. The Midoux number (*Mi*), derived from the Johanson model, was used to predict the ribbon solid fraction at-gap $(SF_{Mi}$). The measured $SF_{gap}$ and the predicted $SF_{Mi}$ lead to a prediction accuracy (*PA*) of the Midoux number. The results are highly dependent on the material used and the applied $P_{max}$. Higher plasticity of the material leads to a reduction in $SF_{ribbon}$ and granule size with increasing *RS*. However, this effect can be overcome or reduced by adjusting $P_{max}$ above the yield pressure of the used material. These results allow for higher roll speeds as a potential upscaling method in roll compaction. On the other side, the *PA* of the Midoux number was also reduced with increased *RS* for MCC and had no effect for lactose. Thus, *RS* seems to be an important factor in the prediction of roll compaction processes and prediction models should include *RS* as a parameter to improve their accuracy.

## 1. Introduction

### 1.1. Roll Compaction/Dry Granulation

**Figure 1:**
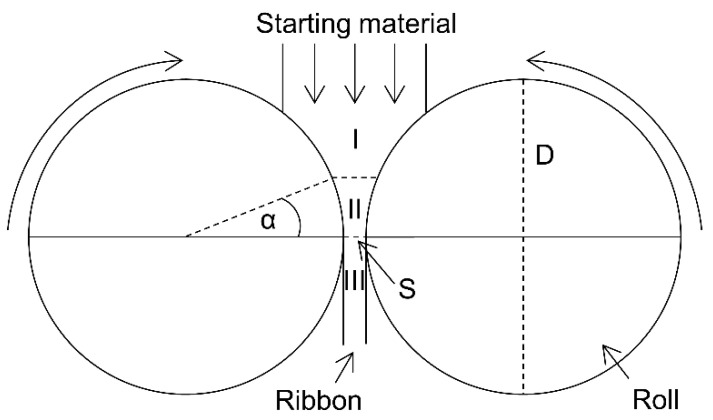
Schematic presentation of roll compaction with slipping zone (I), compaction one (II), release zone (III), roll diameter (*D*), gap width (*S*) and nip angle (*α*).

Roll compaction/dry granulation (*RCDG*) is widely used in pharmaceutics due to its many advantages. It enables continuous manufacturing, is suitable for heat and moisture sensitive materials, reduces dust generation/segregation of powder blends and increases bulk density [1]. Various types of roll compactors are available on the market. They differ in the position of the rolls, the roll geometries, such as the diameter (*D*) and width (*W*), the roll surface, the control mode (gap controlled or screw controlled) and the used sealing system. However, the compaction process is similar for all types. The powder is transported to the rolls via screws. In the slipping zone (I), the powder slides over the counter rotating rolls while being deaerated (Figure 1). The beginning of the compaction zone (II) is determined by the nip angle (*α*) where the powder remains attached to the roll surface and is further transported in the direction of the gap width (*S*), the minimum distance between the rolls. The powder is compacted into ribbons with a certain specific compaction force (*SCF*), which corresponds to the compaction force in kN normalized to the roll width (*W*) in cm. The maximum ribbon solid fraction ($SF_{gap}$), which is reached at the minimum distance between the rolls, is correlated to the degree of densification [2]. The ribbons are released (III), undergoing elastic recovery to reach the ribbon solid fraction ($SF_{ribbon}$) [3] and milled into granules that can be further processed into tablets. The $SF_{ribbon}$ as a key critical quality attribute (CQA) influences the granule size distribution and thus the tabletability [4]. The tabletability can be defined as the ability of a powder to be transformed into tablets with a certain strength under prescribed pressures [5]. A higher *SCF* and lower gap width lead to higher $SF_{ribbon}$ and thus to larger granules sizes [6].

Roll speed (*RS*) as a process parameter in *RCDG* is controversially discussed in the literature. Souihi et al. showed that *RS* has no significant effect on the $SF_{ribbon}$, but a significant negative effect on *D50* (*p* = 0.0128). In addition, higher RS resulted in an increase in granule throughput and a nonsignificant trend (*p* = 0.058) towards improved tabletability. This was explained by the shorter dwell time [6]. As the used materials were predominantly brittle, no reduction in tabletability was observed due to granule hardening. Granule hardening describes the resistance of particularly plastically deformable materials for further deformation after previous compression, e.g., during roll compaction. Nesarikar et al. worked with an instrumented roll compactor and demonstrated that the RS had no significant influence on the maximum pressure $(P_{max}$), which is reached during roll compaction near the gap width. Furthermore, it had no influence on the resulting $SF_{ribbon}$. The authors used a formulation containing microcrystalline cellulose (*MCC*) and anhydrous lactose in a 1:1 ratio and concluded that the effect of *RS* on the ribbon properties was expected to be minimal due to the brittleness of the powder blend. For blends with predominantly plastic or elastic materials, the study needs to be renewed [7]. This was underlined by Al-Asady et al. who compacted only *MCC* at constant hydraulic pressure and roll gap using a gap-controlled roll compactor with different roll speeds from 3 to 7 rpm. Ribbon hardness decreased with increasing RS at all roll angle positions (θ). θ marks the angle between the neutral angle, which can be assumed is at the minimum roll gap and one predefined position on the roll surface, e.g., an installed pressure sensor [8]. Alongside decreasing ribbon hardness, *α* was reduced from 26 to 9°, resulting in a decrease in ribbon tensile strength ($TS_{ribbon}$), increase percentage of fines and lower $SF_{ribbon}$ [9]. Zhang et al. investigated the ribbon density distribution of *MCC* ribbons with terahertz pulsed imaging and observed also a reduction of $SF_{ribbon}$ with increasing *RS* [10]. However, so far, a systematic investigation of *RS* at different *SCF*, considering material properties on the ribbon and granule properties, is missing and will be the aim of this work.

The main disadvantage of *RCDG* is a partial loss of tabletability, which can be explained by two main mechanisms.

1. The granule hardening for mainly plastic deformable materials;
2. The particle size enlargement and decrease in surface binding area for tableting [5].

For a blend containing 60% MCC and 40% acetylsalicylic acid, a strong correlation was observed for reduced tabletability with increased *D50* [11]. It is well known that higher densification due to higher *SCF* leads to an increase in particle size [1]. In this study, particle size enlargement was excluded as a factor by sieving all granules and tableting the same granule size fraction in all experiments. This allows for an investigation at only the effect of *RS* and $P_{max}$ on the tabletabilty. Reduced dwell time at higher RS could lead to improved tabletability as plastic deformation is time dependent.

### 1.2. Models for the Prediction of Roll Compaction Processes

$SF_{gap}$*, S*, *SCF* and $P_{max}$ are key parameters in roll compaction. These parameters determine *CQAs* as granule size distribution and tabletability. Therefore, many approaches have been postulated to predict those parameters: Finite element analysis [12], thin layer model [13], slab method [8] or hybrid modeling [14]. Johanson’s rolling theory for granular solids [15] provides a mathematical approach to predict $SF_{gap}$ using roll geometries (*D*,*W*), process parameters (roll force, $P_{max}$*, S, α*) and material properties (wall/internal friction and compressibility). The practical relevance of Johanson’s model is limited due to the difficulties of measuring the nip angle and wall/friction angle in a laboratory. However, it forms the basis of many simplified or modified models [16,17,18,19,20]. Sousa et al. introduced the dimensionless Midoux number (*Mi*) (Equation (1)) [21]. To get the Midoux number, assumptions must be made.

1. The powder behaves as a solid body being deformed between the counter rotating rolls;
2. The deformation takes place only in one axial direction and can be expressed as uniaxial compression;
3. The mass flow between the rolls in steady state is constant.

Finally, the Midoux number relates $P_{max}$ to the compressibility index (*K*) (Section 2.2.3), the only factor which needs to be determined beforehand, with $P_{\alpha }$ and $\rho_{\alpha }$ to predict the maximum ribbon density at-gap ($\rho_{Mi}$). $P_{\alpha }$ and $\rho_{\alpha }$ are the pressure and powder density at the nip angle. When the Mi is held constant, it leads to a constant $SF_{gap}$. Therefore, the *Mi* can be easily used for equipment transfer and upscaling (Equation (2)). However, the Johanson rolling theory and all the derivatives, including the Midoux number, ignore the roll speed to calculate $P_{max}$ and predict $SF_{gap}$. Therefore, all these models suppose that the change of roll speed has no effect on the $SF_{gap}$ prediction. On the other hand, Sousa et al. noted that increased roll speed could weaken the predictive accuracy of the *Mi* model [21]. Based on this, the aim of this study was to experimentally prove whether the prediction of the *Mi* is really independent of the *RS* using materials with different deformation properties.

(1) $$\frac{P_{max}}{P_{\alpha }}=Mi=\frac{2SCF}{D\rho_{\alpha }}\times \sqrt{\frac{2K}{\pi S/D}}={\left(\frac{\rho_{Mi}}{\rho_{\alpha }}\right)}^{K}$$

(2) $$P_{max}=\frac{2SCF}{D}\times \sqrt{\frac{2K}{\pi S/D}}$$

## 2. Materials and Methods

### 2.1. Materials

The microcrystalline cellulose (*MCC*, Vivapur 102, JRS Pharma, Troisdorf, Germany) was selected as a plastically deformable material and lactose (FlowLac 100, Meggle, Wasserburg am Inn, Germany) as a more brittle material. Lactose was blended with magnesium stearate (Parteck LUB MST, Merck, Darmstadt, Germany) before tableting (Section 2.7). All materials were stored at 21 °C and 45% relative humidity under controlled conditions at least one week before use to allow for equilibration. All prepared ribbons, granules and tablets were also stored for at least one week under these controlled conditions prior to analysis.

### 2.2. Characterization of Raw Materials

#### 2.2.1. Particle Density

The particle density ($\rho_{0}$) of MCC and lactose was determined using an AccuPyc 1330 helium pycnometer (Micromeritics, Norcross, GA, USA) equipped with a 2.5 cm^3^ chamber. Measurements were done in triplicate at constant temperature of 25 ± 1 °C.

#### 2.2.2. Particle Size Distribution

For particle size measurement, dynamic image analysis using Camsizer XT (Retsch, Haan, Germany) with X jet mode was used to ensure disaggregation of agglomerates. The dispersion pressure was 0.4 bar for each run. Powder sampling was done using a rotary sampler (PT 100, Retsch, Haan, Germany). Valid measurements included at least 1,000,000 particles. The *D10*, *D50* and *D90* quantiles of the Q3 distribution of the xc min diameter were used for evaluation. The xc min diameter represents the shortest chord out of the measured set of maximum chords xc. All measurements were performed in triplicates.

#### 2.2.3. Compressibility Index

The compressibility index (*K*), which was used to calculate $P_{max}$ and $SF_{Mi}$, was determined on a Styl’One Evolution (Medelpharm, Beynost, France) equipped with 11.28 mm flat-faced Euro B punches. Tablets were compressed using five tableting pressures between 25 MPa and 250 MPa. At each pressure, 10 tablets were produced and measured. *K* was determined using the slope of the regression between the ln of the in-die tablet density ($\rho_{in-die}$) and the ln of the tableting pressure [18].

#### 2.2.4. Yield Pressure

The yield pressure of MCC and lactose was calculated as the inverse of the Heckel constant using the data of Section 2.2.3. The Heckel constant was determined as the slope of the linear regression between the tableting pressure and the negative ln of the in-die tablet porosity ($\varepsilon_{in-die}$) (Equation (3)). The yield pressure is a surrogate parameter for describing the deformation behavior of materials under pressure. A low yield pressure is associated with plastically deformable materials and higher yield pressures with more brittle materials. To determine $\rho_{in-die}$ (Equation (4)), 10 tablets were weighed using the automatic tablet tester Smart Test 50 (Dr. Schleuniger Pharmatron, Solorhurn, Switzerland). The corrected tablet thickness at maximum force (*h*) and the tablet radius (*r*) were used to calculate the in-die volume ($V_{in-die}$) of each tablet (Equation (5)).

(3) $$\varepsilon_{in-die}=1-\frac{\rho_{in-die}}{\rho_{0}}$$

(4) $$\rho_{in-die}=\frac{m}{V_{in-die}}$$

(5) $$V_{in-die}=\pi *r^{2}*h$$

### 2.3. Roll Compaction

#### 2.3.1. General Settings

A roll compactor BRC25 (L.B. Bohle Maschinen + Verfahren GmbH, Enningerloh, Germany) was used in gap-controlled mode to produce all ribbons. The roll compactor was equipped with knurled rolls of 250 mm *D* and 25 mm *W* and a hybrid sealing system. *S* was kept constant at 2.0 mm for all experiments. The speed ratio between auger and tamping screw was set to 160%. MCC ribbons were compacted at four different *SCF*: 2.9, 4.0, 5.8 and 7.6 kN/cm. As described in Section 2.3.2, these *SCFs* can be converted into $P_{max}$ values, resulting 41, 56, 81 and 106 MPa. The $P_{max}$ values for MCC compaction were chosen so that two pressures were below and two were above the yield pressure of 65 MPa. Accordingly, five $P_{max}$ values: 66, 98, 131, 161 and 193 MPa (SCF: 3.5, 5.2, 7.0, 8.6, 10.3 kN/cm) were selected for lactose, with one value added directly at the yield pressure of 131 MPa. For each $P_{max}$, the roll speed (*RS*) varied between 1.0 rpm and 10.0 rpm. Ribbons were collected for one minute after the process reached steady state conditions ($\Delta SCF\;\pm \;0.1\;\frac{kN}{cm}\;\mathrm{and}\;\Delta S\;\pm \;0.1\;\mathrm{mm}$). All runs were performed in triplicate.

#### 2.3.2. Calculation of $P_{max}$

For both materials, *MCC* (Table 1) and lactose (data not shown), $P_{max}$ was calculated for each applied parameter setting using the determined *K* values for MCC 3.84 and lactose 6.90 (Section 1.2, Section 2.2.3 and Section 2.3.1). Under the assumption of the used model $P_{max}$ being independent of *RS* because *RS* is not included as factor in the *Mi*, the calculated $P_{max}$ values were constant across all roll speeds (Table 1).

#### 2.3.3. Dwell Time in Roll Compaction

**Figure 2:**
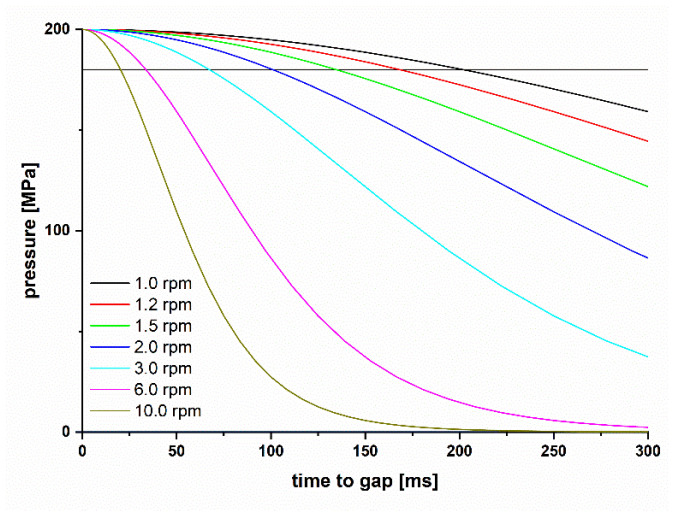
Calculated pressure-time curve for roll compaction (D=250 mm) of MCC at different RS from 1.0 to 10.0 rpm and a fixed $P_{max}$ of 200 MPa. The dwell time is marked as the intersection of the horizontal black line at 180 MPa (90% of $P_{max}$) and the pressure curves.

The dwell time (*DT*) in roll compaction depends on the *RS* (Figure 2). By plotting the pressure-time curve in roll compaction, it was possible to define the dwell time at the point in time before $P_{max}$ is reached (200 MPa in this example) at which the pressure is above 90% of the maximum pressure. The time point is marked as the intersection of the pressure-time curve with the horizontal black line. The pressure curve after passing the gap due to relaxation is ignored in this study for the definition of the *DT*. The pressure-time curve is derived by calculating the pressure at different angles *θ* (Equation (6)) [18] in combination with the angle velocity at different roll speeds. The calculated pressure depends on the compressibility of the used material and therefore the DT at the same RS is different for *MCC* and lactose [17]. The applied roll speeds of 1.0, 1.2, 1.5, 2.0, 3.0 and 6.0 rpm were chosen to have equidistant *DT* steps (Table 2). Further, 10.0 rpm was chosen as the highest roll speed feasible in this setup.

(6) $$P\left(\theta \right)=P_{max}\times {\left[\frac{\frac{S}{D}}{\left(1+\frac{S}{D}-\cos\left(\theta \right)\right)\times \cos\left(\theta \right)}\right]}^{K}\times \cos\left(\theta \right)$$

### 2.4. Characterization of Ribbons

#### 2.4.1. At-gap Ribbon Solid Fraction Measurement ($SF_{gap})$

The at-gap density ($\rho_{gap}$) was measured using the ribbon mass (*m*) and the calculated ribbon volume (*V*) (Equation (7)), which passed the S at a roll width (*W*) in a time period (*t*) of one minute at a given *RS* [13]. The $\rho_{gap}$ (Equation (8)) was used to determine the $SF_{gap}$ (Equation (9)). All measurements were conducted in triplicate.

(7) $$V=S\times W\times \pi \times \left(D+\frac{S}{2}\right)\times RS\times t$$

(8) $$\rho_{gap}=\frac{m}{V}$$

(9) $$SF_{gap}=\frac{\rho_{gap}}{\rho_{0}}$$

#### 2.4.2. Prediction Accuracy (*PA*) of the at-gap Ribbon Solid Fraction Using the Midoux Number

The Midoux number was used to estimate $SF_{Mi}$, the predicted solid fraction at-gap, by referring $\rho_{0}$ to the predicted maximum ribbon density at-gap ($\rho_{Mi}$) (Equation (10)). By comparing $SF_{Mi}$ and the measured at-gap ribbon solid fraction ($SF_{gap}$) (Section 2.4.1), the prediction accuracy (*PA*) of the Midoux number could be determined (Equation (11)). The *PA* was calculated for each applied setting in roll compaction (Section 2.3.1).

(10) $$SF_{Mi}=\frac{\rho_{Mi}}{\rho_{0}}$$

(11) $$PA=\frac{SF_{gap}}{SF_{Mi}}$$

#### 2.4.3. Powder Pycnometry: Ribbon Solid Fraction Measurement ($SF_{ribbon})$

The GeoPyc 1360 powder pycnometer (Micromeritics, Norcross, USA) was used to determine the ribbon density ($\rho_{ribbon}$). The measuring chamber with a diameter of 25.4 mm was used with a default conversion factor of 0.5153 cm^3^/mm. The consolidation force was set to 51 N. The entire ribbon width was used to account for the density distribution over the ribbon width [22]. The sample volume was between 15% and 20%. Triplicate measurements were taken including five blank runs. The density was used to calculate the ribbon solid fraction ($SF_{ribbon}$) (Equation (12)). To compare the effect of the *DT* on the $SF_{ribbon}$ at different $P_{max}$, the *SF* at the lowest DT ($SF_{lowest\;DT}$) was divided by the *SF* at the highest DT ($SF_{highest\;DT}$), e.g., $\frac{SF_{20}}{SF_{20o}}$ for MCC to derive the $SF_{coefficient}$ (Equation (13)).

(12) $$SF_{ribbon}=\frac{\rho_{ribbon}}{\rho_{0}}$$

(13) $$SF_{coefficient}=\frac{SF_{lowest\;DT}}{SF_{highest\;DT}}$$

#### 2.4.4. Ribbon Tensile Strength

**Figure 3:**
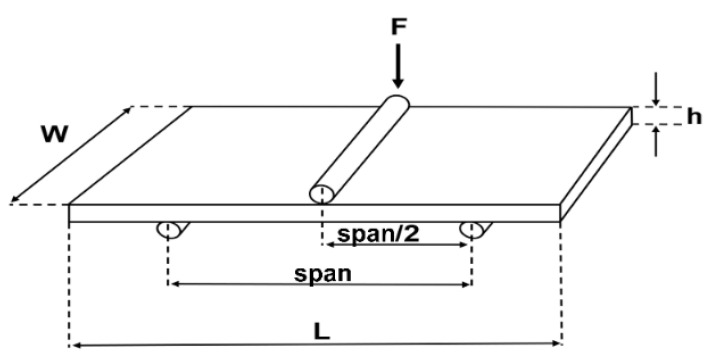
Illustration of a three-point beam fracture test for ribbons with the ribbon width (*w*), ribbon height (*h*), span of loading (*span*), ribbon length (*L*) and force at tensile failure (*F*).

The ribbon tensile strength ($TS_{ribbon}$) was investigated using a Texture analyzer XT2i (Stable Micro Systems Ltd., Godalming, UK) equipped with a three-point beam fracture test (Figure 3) according to Iyer et al. [23]. The force at tensile failure (*F*) was measured as the maximum force of the force-time curve. The span of loading (*span*) is defined as distance between beam 2 and 3 and was set to the maximum available distance of 42 mm. *F* is applied vertically at *span*/2, while *h* and *w* are the thickness and width of the ribbon fragments. h and *w* were measured in triplicate at different positions of the ribbon fragment. The mean was used to determine the $TS_{ribbon}$ (Equation (14)). All measurements were performed in triplicate. It was only possible to measure the $TS_{ribbon}$ of MCC ribbons because the lactose ribbons stuck to the rolls and split during manufacturing. Intact ribbons are required for this type of measurement. Even the use of smooth rolls and external lubrication to prevent sticking could not prevent splitting.

(14) $$TS_{ribbon}=\frac{3*F*span}{2*w*h^{2}}$$

### 2.5. Granulation

A 360° rotating conical sieve (BTS100, L.B. Bohle Maschinen + Verfahren GmbH, Enningerloh, Germany) equipped with 1.5 mm rasp sieve was used for all granulation steps. The rotation speed was kept constant at 200 rpm.

### 2.6. Blending

The lactose powder and granules were blended for 2 min with 0.5% magnesium stearate before tableting using a Turbula type mixer T2C (Willy Bachofen AG, Muttenz, Switzerland).

### 2.7. Tableting

For tableting, a Styl’One Evolution (Medelpharm, Beynost, France) equipped with 11.28 mm flat-faced Euro B punches was used. To exclude the effect of particle size increase with higher SCF, the granules were sieved with an automatic sieve shaker (AS 200 control, Retsch, Haan, Gemany) equipped with sieves of 1400, 1000, 715, 315 and 200 µm. The amplitude was set to 0.5 mm and granules were collected after 3 min. The 315–715 µm fraction was used for tableting in all experiments. Die filling was done manually due to the limited material available. Six tablets of 300 mg each were produced using five tableting pressures from 50 to 250 MPa for all combinations of $P_{max}$ and *RS*.

### 2.8. Characterization of Tablets

Six tablets from each batch were characterized using an automatic tablet tester (Smart Test 50, Dr. Schleuniger Pharmatron, Solothurn, Switzerland). Tablet geometry (*d*, *h*) and fracture force (*F*) were measured to calculate the tensile strength ($TS_{tablet}$) (Equation (15)). The tabletability at different $P_{max}$ and *DTs* were compared.

(15) $$TS_{tablet}=\frac{2\times F}{\pi \times d\times h}$$

## 3. Results and Discussion

### 3.1. Influence of the Roll Speed on the Prediction Accuracy (PA) of the Midoux Number

**Figure 4:**
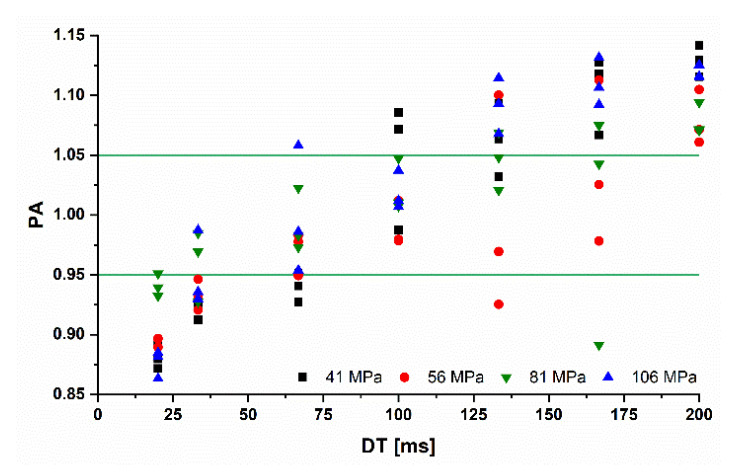
Prediction accuracy (*PA*) for MCC ribbons with changing dwell times from 20 to 200 ms; individual values; n=3; green horizontal bars = *PA* of 1.00 ± 0.05.

**Figure 5:**
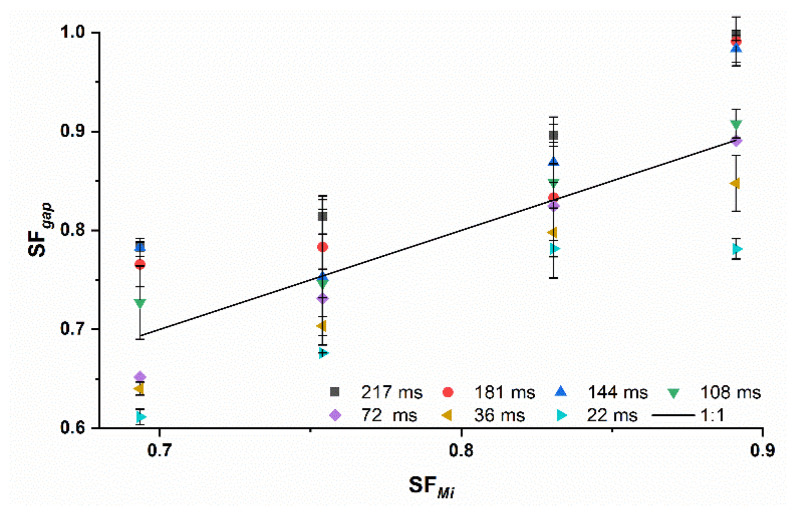
Measured $SF_{gap}$ off *MCC* ribbons with changing dwell times compared to the predicted ribbon solid fraction of the Midoux number $SF_{Mi}$ (solid black line); $\bar{x}\pm s$; n=3.

The *PA* of the Midoux number for *MCC* ribbons is dwell time dependent. The best prediction was obtained at *RS* of 2 and 3 rpm, corresponding to *DTs* of 108 and 72 ms (Figure 4 and Figure 5), which are commonly used in roll compaction [9,24,25,26]. These authors used different roll compactors with *D* of 50–120 mm (250 mm in this study). Due to the influence of the *D* on the *DT*, their resulting *DTs* are not identical to 108/72 ms using the same *RS*.

However, in this study, 2 and 3 rpm led to prediction accuracies over all $P_{max}$ of 1.02 ± 0.03 and 0.98 ± 0.04, respectively (arithmetic mean ± standard deviation:$\;\bar{x}\pm s$). These predictions were closest to the 1:1 line, which is the prediction of the *Mi* number. Higher roll speeds led to an overestimation of the $SF_{gap}$ due to the lower actual $P_{max}$ (Section 3.2) and lower *RSs* to underestimation (Figure 5). Increased roll speed might reduce the $P_{max}$ [8], which leads to reduced compaction and lower $SF_{gap}$. Lower $SF_{gap}$ reduces the *PA* (Equation (11)). For *RS* lower than 2 rpm, the $P_{max}$ might be above the predicted value of the *Mi* and leads therefore to higher $SF_{gap}$ than predicted. The *PA* increases above 1.

**Figure 6:**
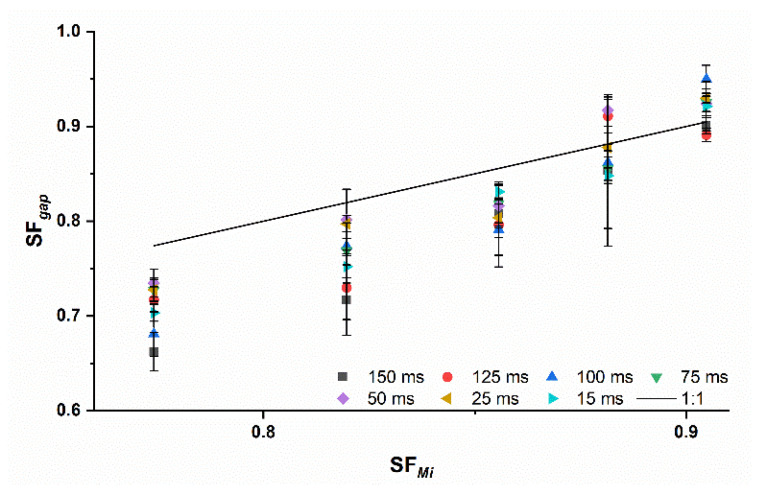
Measured $SF_{gap}$ off lactose ribbons with changing dwell times compared to the predicted ribbon solid fraction of the Midoux number $SF_{Mi}$ (solid black line); $\bar{x}\pm s$; n=3.

This underlines the importance of considering the roll speed for the prediction of the $SF_{gap}$. Including *RS* as a factor in the Midoux number would probably better the *PA* if different roll speeds were used. For more brittle materials such as lactose, no clear trend was observed for the dependency of the *DT* on *PA* (Figure 6).

### 3.2. Influence of the Roll Speed on the $SF_{ribbon}$

**Figure 7:**
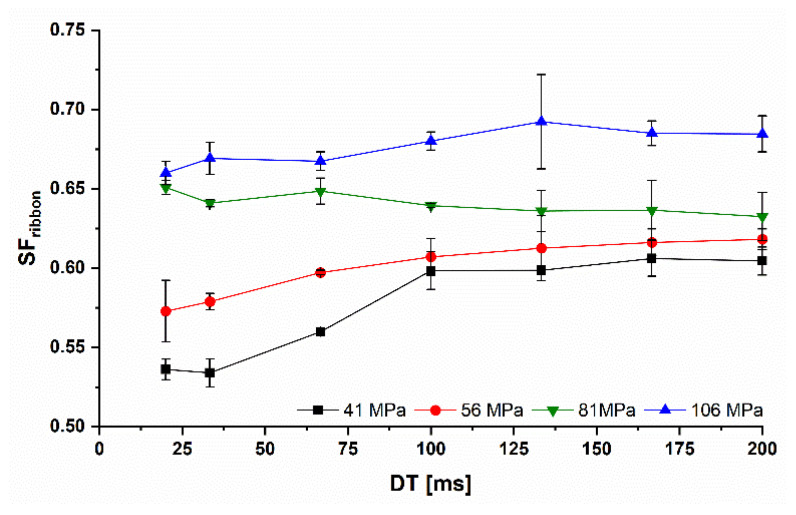
Dwell time dependent *SF* of *MCC* ribbons at different $P_{max}$ from 41 to 106 MPa; $\bar{x}\pm s$; n=3.

*The*$SF_{ribbon}$ increased when $P_{max}$ was increased due to the higher compaction of the ribbons. As explained in Section 2.3.1, two $P_{max}$ values were chosen below and above the yield pressure of *MCC*, which was determined to be 65 MPa. For $P_{max}$ below the determined yield pressure, the $SF_{ribbon}$ decreased with a reduction of the dwell time below 100 ms (Figure 7). Comparing the $SF_{coefficent}$ for $P_{max}$ of 41 and 56 MPa, 0.89 and 0.93, the reduction of $SF_{ribbon}$ was lower for 56 MPa. The decrease depends therefore on the used $P_{max}$. The higher the $P_{max}$, the closer $SF_{20}$ is to $SF_{200}$ and $SF_{coefficent}$ tends to 1. The calculated $P_{max}$ using the *Mi* number was independent of the *RS* (Section 1.2 and Section 2.3.2). However, Patel et al. showed that an increase in *RS* leads to a decrease in maximum pressure and nip angle [8]. This can explain why the densification was reduced at lower *DTs*. The calculated $P_{max}$ may not be the actual $P_{max}$ when the *RS* increases. The area under the pressure-angle curve is smaller, resulting in lower compaction. However, this hypothesis needs to be proven using pressure sensor instrumented rolls.

At $P_{max}$ of 81 and 106 MPa, no or only a slight reduction of the $SF_{ribbon}$ could be observed showing $SF_{coefficent}$ of 1.03 and 0.96. This could be due to the fact that the reduced $P_{max}$ at increased *RS* is still around or above the yield pressure of *MCC*. Predominantly plastic deformation leads to comparable $SF_{ribbon}$ with only small time-dependent reduction. In order to keep the ribbon solid fraction as constant as possible, while working with increased *RS*, $P_{max}$ should be increased above the yield pressure of the used formulation. This could be a solution for using *RS* as a potential upscaling tool in *RCDG*.

**Figure 8:**
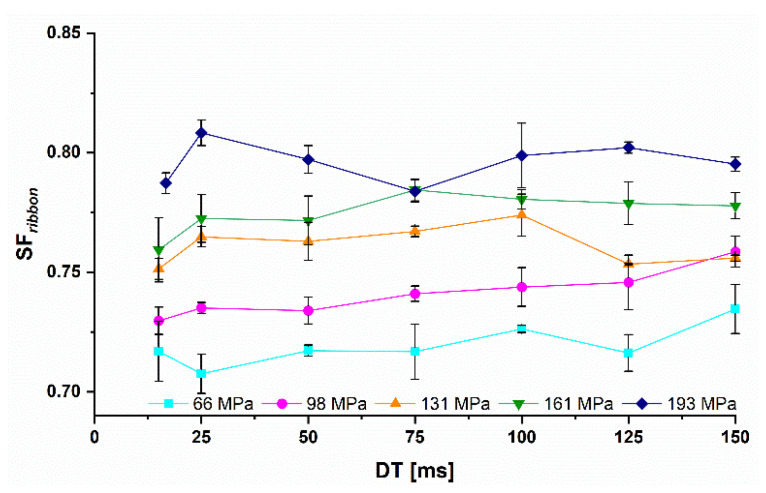
Dwell time dependent *SF* of lactose ribbons at different $P_{max}$ from 66 to 193 MPa; $\bar{x}\pm s$; n=3.

Similar to *MCC*, the $SF_{ribbon}$ of lactose ribbons increased as expected with higher $P_{max}$. However, for lactose, only a negligible decrease in the $SF_{ribbon}$ was observed at higher *RS* and lower *DT* (Figure 8). The $SF_{coefficent}$ at all $P_{max}$ were in the range of 0.96 to 0.99. This could be explained by the fact that only small proportions of max. 10–15% of the lactose grade are amorphous and exhibit a plastic deformation behavior [27]. The predominantly brittle behavior of lactose, confirmed by the higher yield pressure of 131 MPa, could be more independent with respect to the $SF_{ribbon}$ when the *RS* was increased. The potentially reduced pressure seems to have a little or no influence on the $SF_{ribbon}$.

In summary, the effect of the *RS* on the $SF_{ribbon}$ is hardly influenced by the presented material deformation properties. The plastically deformable material showed a lower $SF_{ribbon}$ for shorter *DT* at $P_{\max}$ below the yield pressure, while the more brittle material showed almost no effect on their $SF_{ribbon}$ regardless of $P_{\max}$. Higher $P_{\max}$ stabilized the $SF_{ribbon}$ even for plastically deformable materials like *MCC*. The upscaling for more brittle materials is therefore more independent of the roll speed than for plastic materials. Higher *RS* can be used without a sharp drop in the ribbon solid fraction.

### 3.3. Influence of the Roll Speed on the Granule Size

**Figure 9:**
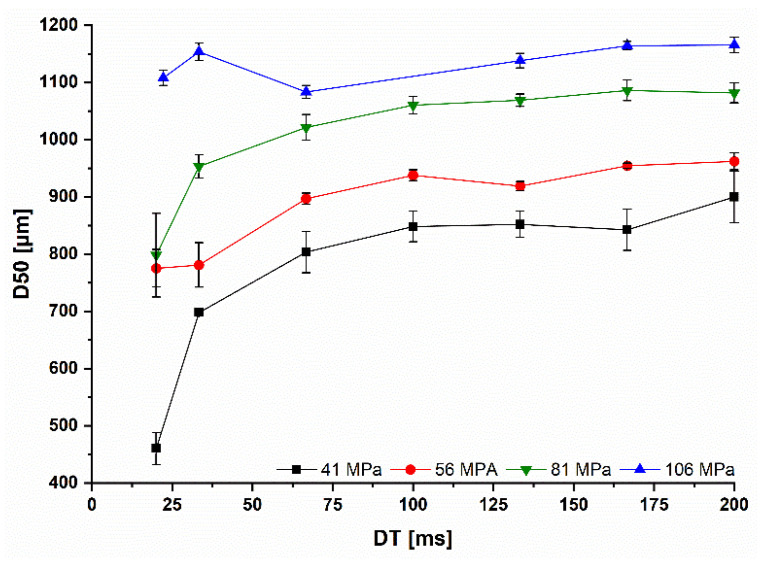
Influence of the dwell time on the median granule size *D50* of *MCC* granules at different $P_{max}$ from 41 to 106 MPa; $\bar{x}\pm s$; n=3.

**Figure 10:**
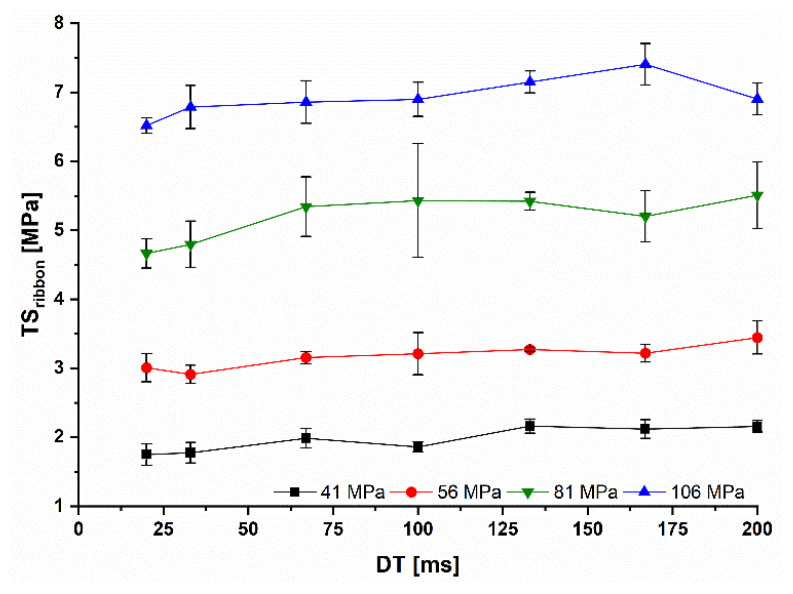
Ribbon tensile strength at different $P_{max}$ and RS of MCC ribbons; $\bar{x}\pm s$; n=3.

The particle size of the MCC granules, represented as the *D50* value, increased with higher $P_{max}$ due to higher $SF_{ribbon}$ (Figure 9). The *D50* value was reduced for 41 and 56 MPa with reduced *DT*. This goes alongside with the reduced $SF_{ribbon}$ below 100 ms (Figure 5). The highest reduction was accompanied by the highest reduced $SF_{ribbon}$ at 41 MPa and 20 ms (Figure 7 and Figure 9). Surprisingly, the *D50* at 81 MPa declined with higher *RS*, although a decrease in the $SF_{ribbon}$ was not observed. This can be explained by the decrease of $TS_{ribbon}$, which is most pronounced at $P_{max}$ of 81 MPa with a $TS_{ribbon}$ decrease of 0.81 MPa between 200 and 20 ms (Figure 10). However, the reason for this is not yet clear. At all other $P_{max}$, the $TS_{ribbon}$ decreased between 0.38 and 0.44 MPa comparing *DTs* of 200 and 20 ms.

Only at the highest $P_{max}$, the particle size remained almost the same with a maximum reduction of 70 µm, which is consistent with the more constant $SF_{ribbon}$. The same tendency could be observed for *D10* and *D90* values. However, the reduction in the particle size, which was a maximum of 60 µm for both *D10* and *D90*, was much smaller than for *D50*. This small reduction in *D10* and *D90* is not expected to have large effect on the tabletability or flowability. The work space for upscaling by increasing *RS* should be above the yield pressure of the powder blend with some safety level to ensure more constant $SF_{ribbon}$, $TS_{ribbon}$ and *D50*. Working with increased *RS* at lower $P_{max}$ otherwise leads to a high reduction in the *D50*, which is associated with poorer flowability and altered tableting properties.

**Figure 11:**
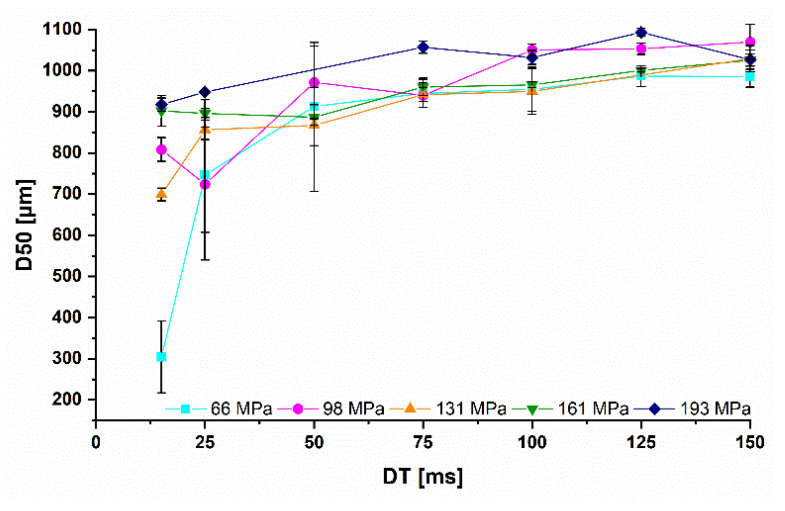
Influence of the dwell time on the median granule size of lactose granules at different $P_{max}$ from 66 to 193 MPa; $\bar{x}\pm s$; n=3.

The influence of the $SF_{ribbon}$ for lactose on the particle size could not be shown as for *MCC* (Figure 8 and Figure 11). For the three highest *DTs*, the *D50* was almost independent of the $SF_{ribbon}$. Overall, the particle size was reduced in all cases with higher *RS*. Especially at 6 and 10 rpm, the *D50* dropped for $P_{max}$ values of 66, 98 and 131 MPa. Above 131 MPa, the decrease in particle size was smaller.

### 3.4. Impact of the Roll Speed on Tabletability

**Figure 12:**
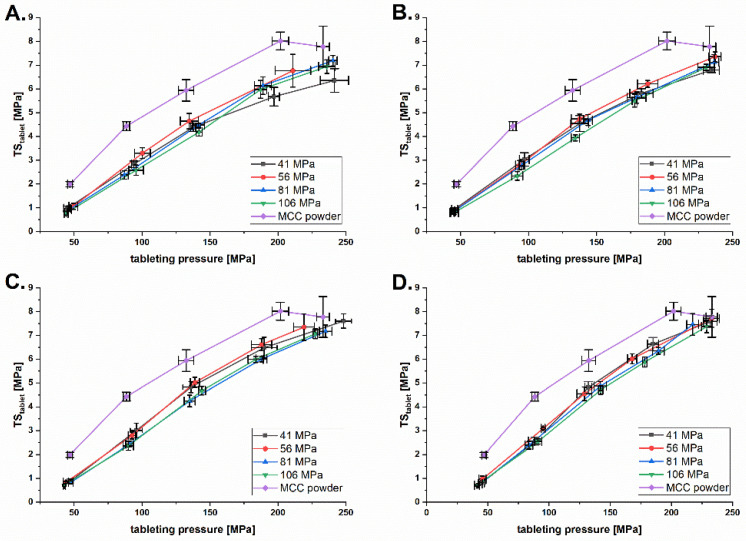
Tabletability plot of MCC granules at *DTs* of 200 ms (**A**), 133 ms (**B**), 67 ms (**C**) and 20 ms (**D**) and $P_{max}$ of 41–106 MPa.

The tabletability of MCC granules was affected by the granule hardening due to previous plastic deformation during roll compaction under high pressure (Figure 12). Surprisingly, the effect was more pronounced at lower *DTs* (Figure 12C,D) and less at higher *DTs* (Figure 12A,B). The MCC powder always has a higher $TS_{tablet}$, which can be explained by the higher binding area at a particle size of 163 ± 1.5 µm and no upstream compaction step. The overall tabletability of the MCC powder and granules is lower compared to the results of Mosig and Kleinebudde [28]. This can be explained by the higher particle size of the *MCC* powder used on this study. This is in line with the finding that a increase of particle size of *MCC* resulted in lower tabletability of *MCC* powder and corresponding *MCC* granules [29]. In addition, *MCC* 102 powder and granules have the lowest tabletability compared to *MCC* 101 and *MCC* 105 [30]. The reduction in tabletability was not as high as in previous studies [28] because the maximum *SCF* used was 7.6 kN/cm. Mosig and Kleinebudde instead additionally used *SCFs* of 8, 10 and 12 kN/cm. Higher *SCFs* lead to higher reworkability and therefore lower tabletability.

**Figure 13:**
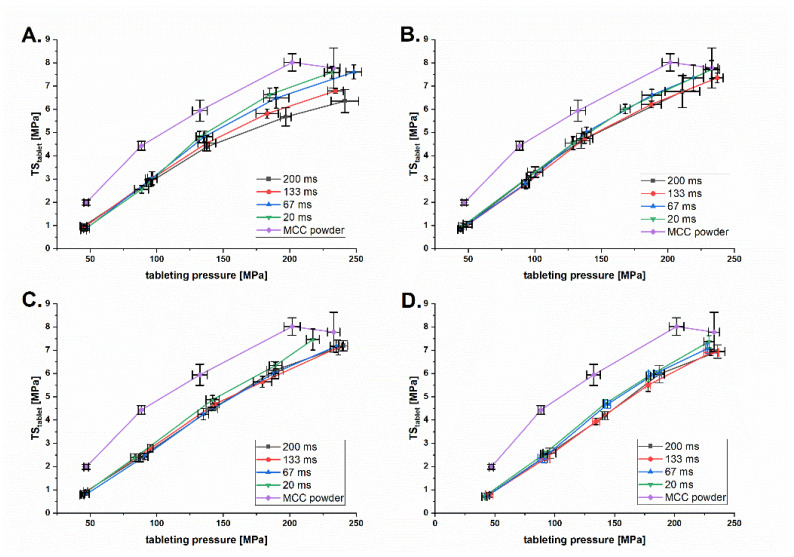
Tabletability plot of *MCC* granules at $P_{max}$ of 41 MPa (**A**), 56 MPa (**B**), 81 MPa (**C**) and 106 MPa (**D**) and *DT* of 20–200 ms.

Decreasing *DT* did improve tabletability (Figure 13). This was most prominent at the lowest $P_{max}$ of 41 MPa (Figure 13A) and is consistent for all higher $P_{max}$. Lower *DT* seems to minimize plastic deformation and therefore increase tabletability. This can be explained by the dwell time dependence of plastic deformation [31]. In summary, roll compaction has a negative effect on the tabletability of *MCC*, which can be balanced by the positive effect of the decreased *DT* on the $TS_{tablet}$.

The tabletability of lactose granules showed no dwell time dependence at the five different used $P_{max}$ (Figure S1). The effect of the $P_{max}$ is contra intuitive, as the tabletability increased with higher $P_{max}$ (Figure S2). Until now, no explanation could be found. Lactose powder with a *D50* of 151 ± 1.5 µm showed a higher binding area and thus a higher tabletability compared to the granular fraction used [5]. The range of $TS_{tablet}$ of the lactose granules is in line with the results of Wu and Sun [32].

## 4. Conclusions

The Midoux number provides a novel and simple way to predict the solid fraction of ribbons at-gap width without investing a lot of time and material. However, until now, the influence of the roll speed on the prediction accuracy has been neglected because the Johanson model and all the derivates, including the Midoux number, did not include the roll speed as a factor on the prediction of the roll compaction process. The roll speed can have an impact on the prediction accuracy of the ribbon solid fraction at-gap ($SF_{gap})$, considering different kind of materials. For a more plastically deformable material, such as microcrystalline cellulose, the prediction accuracy drops from 1.15 to below 0.9, with the highest accuracy between 2 and 3 rpm (0.026 and 0.039 m/s). Thus, roll speed seems to be an important factor in the prediction of roll compaction processes at least for highly plastically deformable materials, and prediction models should include the roll speed as parameter to improve their accuracy. For a more brittle material like lactose, no dependence between the roll speed and the prediction accuracy could be found.

On the other hand, the roll speed can also have an influence on the solid fraction of the ribbons ($SF_{ribbon})$. Microcyrstalline cellulose ribbons showed a decrease of their solid fraction if the maximum roll pressure is below the yield pressure of the material. Above the yield pressure, no or only a small reduction was observed. The same trend could be shown for the corresponding granule size distributions. For a rather brittle material like lactose, the roll speed has a neglectable influence on the ribbon solid fraction. In contrast to the low influence on the ribbon solid fraction, the particle size decreases when maximum roll pressure is below the yield pressure. Above the yield pressure, the particle size decreases less at low dwell times. Thus, the ribbon density is not the only important parameter determining the particle size distribution. Roll compaction has a negative effect on the tabletability due to granule hardening for microcrystalline cellulose. However, on the other hand, the decrease in the dwell time had a positive effect on the tabletability. This may indicate upscaling by increasing the roll speed as a potential new method. Until now, upscaling by increasing the roll speed is not commonly used because of the shown effect on the ribbon and granule properties, which must be taken into account. Higher roll speeds should be used in conjunction with compaction pressures above the yield pressure of the powder blend to ensure consistent critical quality attributes. Furthermore, roll compaction, which followed a loss in tabletability, may be overcome by the positive effect of increased roll speed on the tabletability. Higher roll speeds seem to be a potential way to reduce tabletability issues. In conclusion, further studies should investigate whether increasing roll speed could be a potential upscaling method compared to a standard up-scaling method, e.g., larger roll width.

## Figures and Tables

**Table 1:** Calculated $P_{max}$ for the compaction of *MCC* at different *SCF* and *RS* combinations.

*SCF* [kN/cm]	Gap Width [mm]	*RS* [rpm]	Compressibility Index (*K*)	$P_{max}\;[\mathrm{MPa}]$
2.9	2.0	1.0–10.0	3.84	41
4.0	2.0	1.0–10.0	3.84	56
5.8	2.0	1.0–10.0	3.84	81
7.9	2.0	1.0–10.0	3.84	106

**Table 2:** *DT* in ms for the roll compaction of *MCC* and lactose with different roll speeds.

*RS* [rpm]	*MCC*	Lactose
1.0	200	150
1.2	167	125
1.5	133	100
2.0	100	75
3.0	67	50
6.0	33	25
10.0	20	15

## Data Availability

Data are available on request.

## Supplementary Materials

The following supporting information can be downloaded at: https://www.mdpi.com/article/10.3390/pharmaceutics14112399/s1, Figure S1: Tabletability plot of lactose granules at $P_{max}$ of 66 MPa (A), 98 MPa (B), 131 MPa (C), 161 MPa (D) and 193 MPa (E) and DT of 15–250 ms; Figure S2: Tabletability plot of lactose granules at DTs of 150 ms (A), 100 ms (B), 50 ms (C) and 15 ms (D) and $P_{max}$ of 66–193 MPa. Figure S3: DT dependent $SF_{gap}$ of MCC ribbons at different $P_{max}$; $\bar{x}\pm s$; n=3. Figure S4: DT dependent $SF_{gap}$ of lactose ribbons at different $P_{max}$; $\bar{x}\pm s$; n=3.

## Abbreviations

*I*	Slipping zone
*II*	Compaction zone
*III*	Release zone
*α*	Nip angle
$\varepsilon_{in-die}$	In-die tablet porosity
*θ*	Roll angle
$\rho_{o}$	Particle density
$\rho_{\alpha }$	Particle density at nip angle
$\rho_{gap}$	Density at gap width
$\rho_{in-die}$	In-die tablet density
$\rho_{Mi}$	Predicted density at gap width
$\rho_{ribbon}$	Ribbon density
*CQA*	Critical quality attribute
*D*	Roll diameter
*d*	Tablet diameter
*D10*	10 percent quantile of the granule size distribution
*D50*	Median granule size
*D90*	90 percent quantile of the granule size distribution
*DT*	Dwell time
*F*	Force at tensile failure
*h*	height
*K*	Compressibility index
*L*	Length
*m*	Mass
*MCC*	Microcrystalline cellulose
*Mi*	Midoux number
PA	Prediction accuracy of the Midoux number
$P_{\alpha }$	Pressure at nip angle
$P_{max}$	Maximum roll pressure at gap width
*r*	Radius
*RCDG*	Roll compaction/dry granulation
*RS*	Roll speed
*S*	Gap width: minimum distance between the rolls
*SCF*	Specific compaction force
*SF*	Solid fraction
$SF_{coefficient}$	Ratio between the solid fraction at lowest and at the highest dwell time
$SF_{gap}$	Ribbon solid fraction at gap width
$SF_{highest\;DT}$	Ribbon solid fraction at the highest dwell time
$SF_{lowest\;DT}$	Ribbon solid fraction at the lowest dwell time
$SF_{Mi}$	Predicted ribbon solid fraction using the Midoux number
*span*	Span of loading
*t*	Time
$SF_{ribbon}$	Ribbon solid fraction
$TS_{ribbon}$	Ribbon tensile strength
$TS_{tablet}$	Tablet tensile strength
*V*	Ribbon volume
$V_{in-die}$	In-die tablet volume
*W*	Roll width
*w*	Ribbon width
