# Supplementary material for: Influence of Roll Speed during Roll Compaction and Its Effect on the Prediction of Ribbon Solid Fraction
Source: Pharmaceutics. 2022 Nov 7;14(11):2399. doi: 10.3390/pharmaceutics14112399 (PMC9694291; doi:10.3390/pharmaceutics14112399)
Supplement: Supplementary file 1 [file pharmaceutics-14-02399-s001.zip › pharmaceutics-2000449-supplementary.pdf]

Influence of roll speed during roll compaction and its effect on the prediction of ribbon solid fraction

Martin Lück [1], Matthias De Saeger [1,2] and Peter Kleinebudde [1,*]

Supplementary Materials:

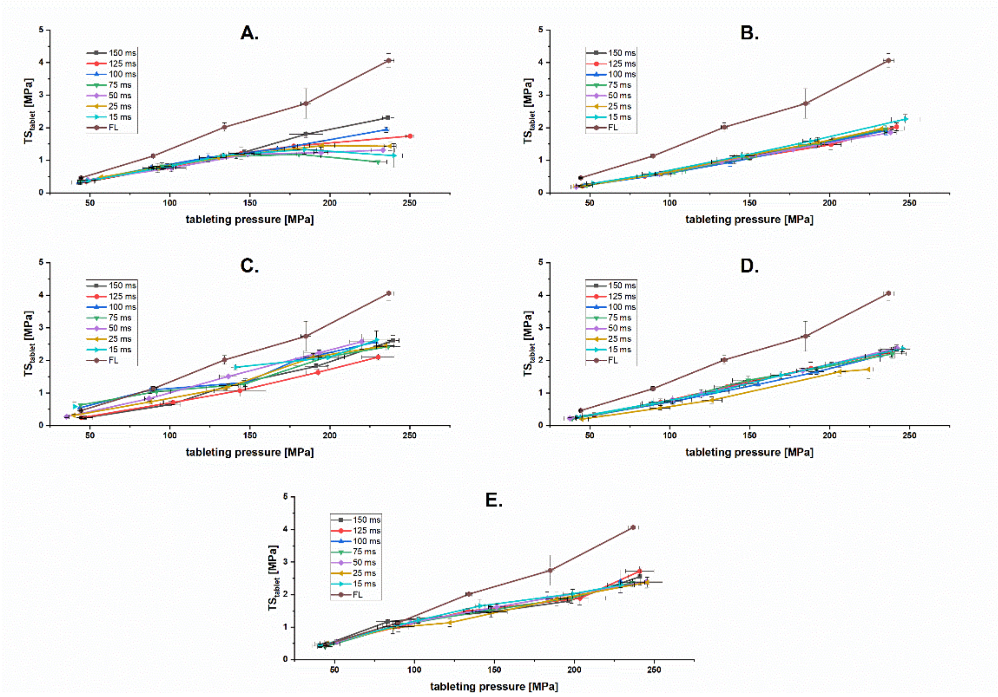

Figure S1: Tabletability plot of lactose granules at P_{max} of 66 MPa (A), 98 MPa (B), 131 MPa (C), 161 MPa (D) and 193 MPa (E) and DT of 15-250 ms.

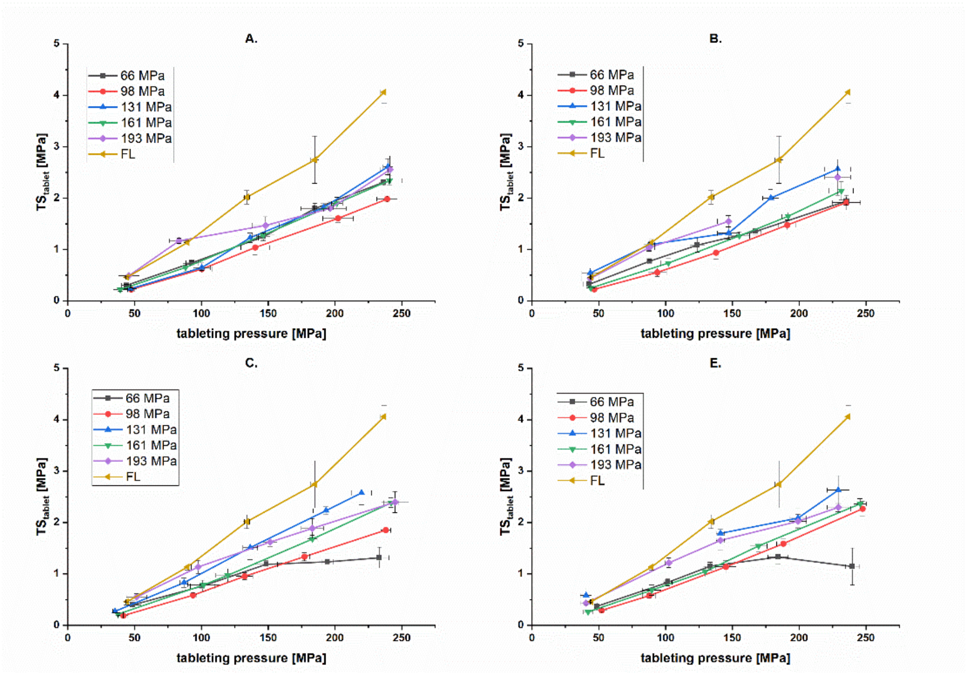

Figure S2: Tabletability plot of lactose granules at DTs of 150 ms (A), 100 ms (B), 50 ms (C) and 15 ms (D) and P_{max} of 66-193 MPa.

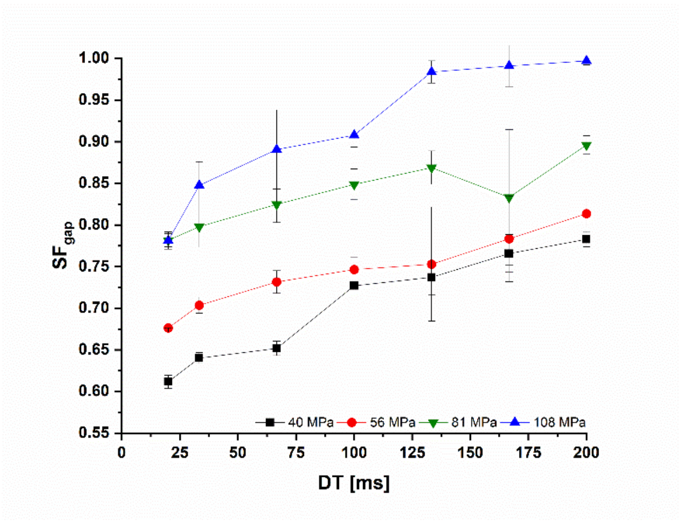

Figure S3: DT dependent SF_{gap} of MCC ribbons at different P_{max}; $\bar{x} \pm s$; $n = 3$.

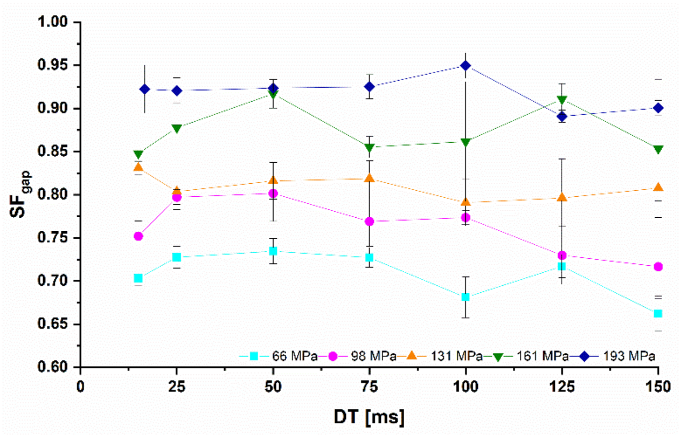

Figure S4: DT dependent SF_{gap} of lactose ribbons at different P_{max}; x̄±s; n=3.
